# Excimer laser for the treatment of incomplete rerepigmentation 1 year after cultured epidermal autograft use for carbon dioxide laser–ablated lesions in patients with stable vitiligo

**DOI:** 10.1016/j.jdcr.2024.02.033

**Published:** 2024-03-15

**Authors:** Hiroshi Kato, Kazuhiro Toriyama, Yuki Enomoto, Yoshifumi Kanayama, Aya Yamamoto, Hideyoshi Sato, Tomoyo Tanaka, Masukazu Inoie, Akimichi Morita

**Affiliations:** a Department of Geriatric and Environmental Dermatology, Nagoya City University Graduate School of Medical Sciences, Nagoya, Japan; b Department of Plastic and Reconstructive Surgery, Nagoya City University Graduate School of Medical Sciences, Nagoya, Japan; c Japan Tissue Engineering Co, Ltd, Gamagori, Japan

**Keywords:** cultured epidermal autograft, excimer laser, laser, minigraft, photpchemothrapy, phooto serapies, surgery, transparantation, Vitiligo

## Introduction

Surgical treatment is used to treat vitiligo vulgaris refractory to conservative treatments such as topical corticosteroids and phototherapy ^1^and the use of procedures, such as 1-mm minigraft therapy and suction blister extravasation, has been reported.^2^^,^^3^ We have previously reported treating such cases using carbon dioxide laser epidermal ablation and cultured epidermal autograft (CEA).^4^ The epidermis of the affected area was removed by superficial ablation with a CO2 laser AcuPluse (Lumenis Be Japan Co, Ltd). Laser irradiation was fully ablative and used SilkTouch Scanner (moderate surface ablation with a double scan) to transpire the epidermis. Cultured epidermis containing melanocytes was prepared by the Green culture method, and the epidermis of the vitiligo area was removed by carbon dioxide laser before transplantation. Although most patients show improvements after CEA, a certain percentage shows insufficient repigmentation. Here, we describe cases of patients with vitiligo in whom excimer laser treatment provided good results after sufficient repigmentation was not achieved after CEA. Although there are several reports on excimer laser irradiation methods, we followed the protocol of Noborio et al.^5^ for excimer laser treatment: minimal blistering dose was measured, 1 minimal blistering dose was applied in a grid pattern each time, and irradiation was performed once a week.

## Case report

### Case 1

**Fig 1:**
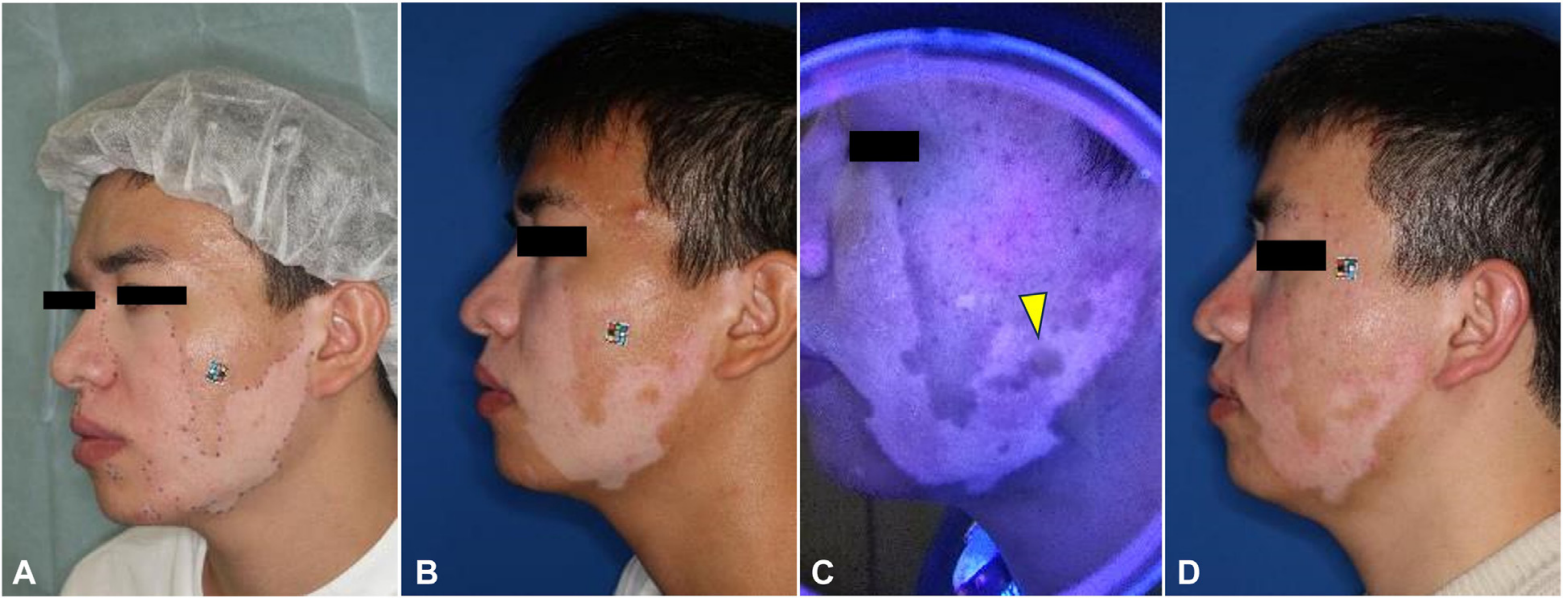
**A,** Clinical photograph prior to CEA. **B,** Clinical photograph 1 year after transplantation. **C,** Wood light photograph taken after 40 excimer laser irradiation sessions Pale repigmentation is seen in some areas of vitiligo, some of which have a tendency to enlarge toward the periphery (*arrowhead*). **D,** Clinical photograph taken after 53 excimer laser treatment sessions. *CEA*, Cultured epidermal autograft.

A 24-year-old man presented to our clinic with increasing vitiligo over the left mandible region since the age of 22 (Fig 1, *A*). After 1 year of treatment with topical corticosteroids and tacrolimus ointment without improvement, it was confirmed that vitiligo had stopped increasing and hence CEA was performed. However, 1 year and 3 months after surgery, pigment regeneration was insufficient (Fig 1, *B*); therefore, excimer laser irradiation was initiated at 150 mJ/cm^2^. Repigmentation was observed in the surrounding area after 40 sessions (Fig 1, *C*) and continued to spread after 53 sessions (Fig 1, *D*).

### Case 2

**Fig 2:**
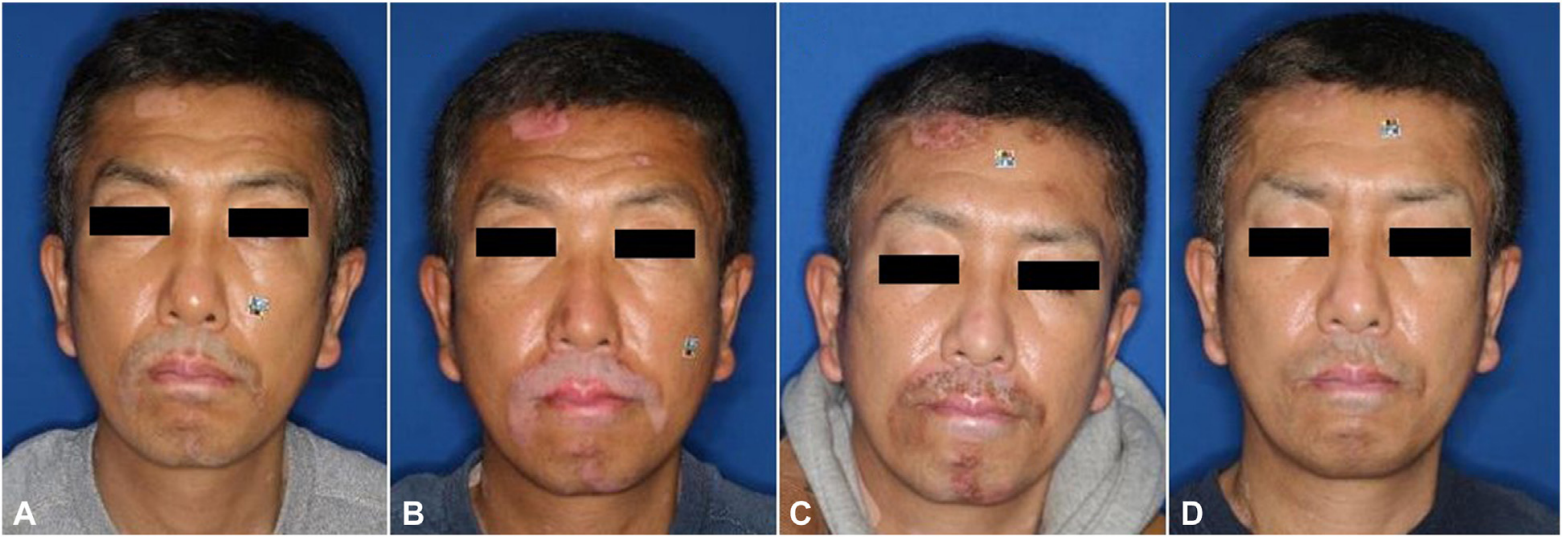
**A,** Clinical photograph before CEA. **B,** Clinical photograph taken 1 year after transplantation. **C,** Clinical photograph taken after 11 excimer laser treatment sessions. **D,** Clinical photograph taken after 50 excimer laser treatment sessions. *CEA*, Cultured epidermal autograft.

A 51-year-old man presented with facial vitiligo that began to develop at the age of 45 (Fig 2, *A*). The lesions did not respond to topical treatment and tended to increase in size progressively. Therefore, autologous cultivated epidermal skin grafting was performed. The skin fixation was satisfactory. However, the repigmentation was not up to the mark, as depicted in Fig 2, *B*. At 1 year and 4 months after the procedure, excimer laser treatment was started at an irradiation dose of 125 mJ/cm^2^; patchy repigmentation was observed after 11 sessions (Fig 2, *C*), and good repigmentation was achieved after 50 sessions (Fig 2, *D*).

### Case 3

**Fig 3:**
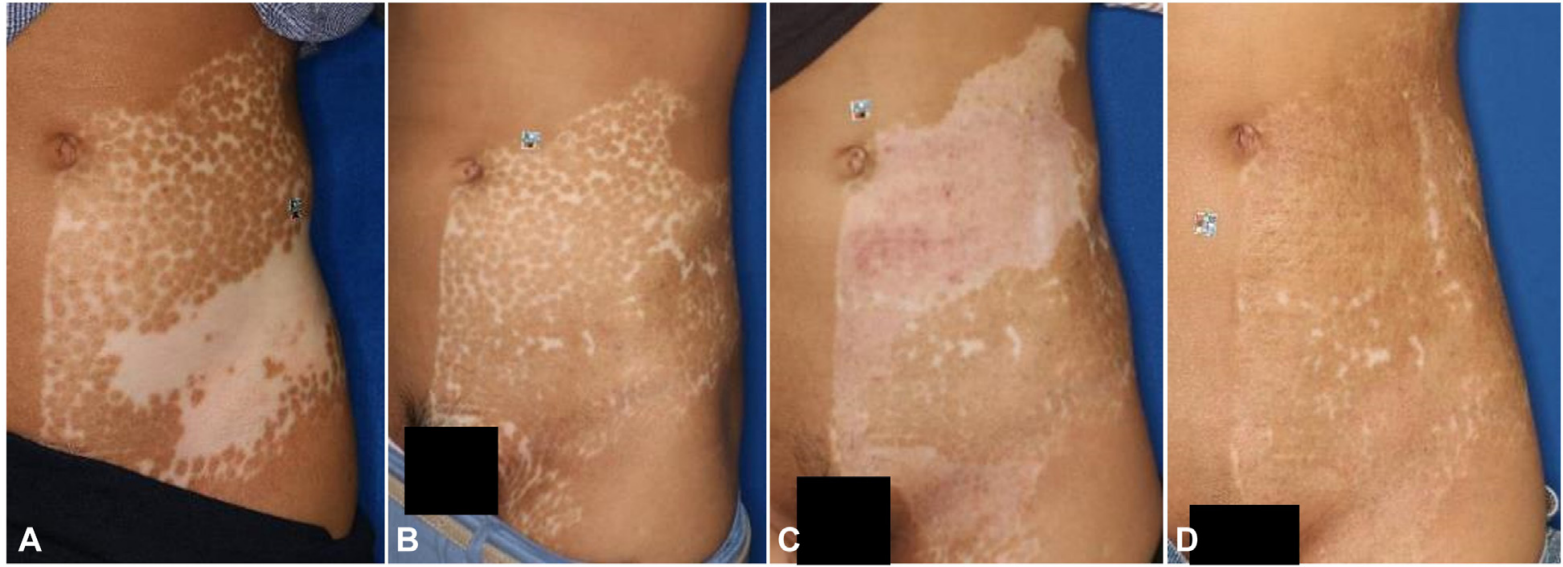
**A,** Clinical photograph before CEA. Punctuate repigmentation is observed after the 1-mm minigraft procedure. Central leukoplakia was not treated using tail punch grafting. **B,** Clinical photograph taken 1 year after CEA of the surrounding punch graft area. The color of the said area is much lighter than that of the surrounding area. The *red dotted line* indicates the graft site. **C,** Clinical photograph taken after 11 excimer laser treatment sessions. Patchy hyper-repigmentation can be seen. **D,** Clinical photograph of the graft site taken after 18 excimer laser treatment sessions. The repigmentation is the same as that in the surrounding area. *CEA*, Cultured epidermal autograft.

A 19-year-old woman had vitiligo on the lateral abdomen, which had begun to develop at the age of 9. She was treated with narrow-band UVB therapy by a local physician at the age of 10 years; however, there was no improvement. Between the ages of 12 and 15, she received multiple 1-mm minigrafts (Fig 3, *A*), and at the age of 16, she underwent partial CEA at her abdomen, which was successful (Fig 3, *B*). Later, at the age of 19, a second graft was applied in the same area where the previous 1 mm minigraft was used. Subsequently, repigmentation was observed; however, it was pale compared with the surrounding skin (Fig 3, *C*). Therefore, 1 year and 5 months after the procedure, excimer laser irradiation was initiated at 125 mJ/cm^2^. After 18 sessions, repigmentation of a similar contrast to the surrounding skin was observed (Fig 3, *D*).

## Discussion

Although topical corticosteroids, topically active vitamin D3, and phototherapy have been used to treat vitiligo with some success, many patients do not respond well to these modalities and require surgical treatment.^6^ Since the treatment of vitiligo with split-thickness skin grafts was first reported in 1947, several surgical treatments have been reported.^7^ Punch grafting was the first treatment reported; however, its indications were limited owing to the deformation of the paving stone change of the skin.^8^ We previously reported a variant of 1-mm minigraft therapy for vitiligo, which can afford repigmentation with minimal scarring.^2^ However, unsatisfactory results have been observed in some cases of segmental vitiligo and many cases of generalized vitiligo.^6^ One study reported that the addition of postoperative narrow-band UVB therapy in refractory cases facilitated repigmentation.^9^ One drawback of 1-mm minigraft therapy is that vitiligo may persist between grafts. Case 3 is a typical example. We previously reported the usefulness of autologous cultivated epidermal grafts in such cases.^4^ Many cases can be cured using this method; however, in a few cases, postoperative repigmentation is insufficient.

The 308 nm excimer laser is useful for vitiligo, and we have seen cases in which conventional narrow-band UVB and excimer light therapies failed to produce results.^10^ Homan et al compared the results of punch grafting for vitiligo with those of excimer laser and narrow-band UVB irradiation. They reported no significant differences in repigmentation; however, the cumulative dose was 71% lower in the excimer laser group.^11^ Al-Mutairi et al reported that postoperative excimer laser treatment of vitiligo treated with split-thickness skin grafts resulted in repigmentation in all patients, and the long-term prognosis was good in all cases.^12^ In addition, it has been shown that better repigmentation can be achieved by using excimer laser after melanocyte stem cell transplantation.^13^

In this study, we performed excimer laser irradiation in patients with inadequate repigmentation after CEA. The irradiation was started more than 1 year after surgery, which was a sufficient time to confirm that the effect of surgery was inadequate. Because some cases of CEA for vitiligo begin to develop hyper-repigmentation several months later, this clinical trial protocol included a 1-year follow-up after transplantation. Therefore, excimer laser treatment was to be initiated 1 year after transplantation. Although the introduction of narrow-band UVB or excimer light in the early postoperative period is thought to improve the prognosis of vitiligo patients, there is a lack of evidence regarding the timing of initiation of laser therapy in the combination of CEA and excimer laser. The aforementioned report on postoperative irradiation was started approximately 2 months after surgery, and the timing of starting irradiation should be discussed in the future. We found evidence supporting the safety and efficacy of excimer laser irradiation after the application of autologous cultivated epidermal grafts. However, it is important to note that this is essentially a case report, and further research is needed to establish the safety and efficacy of excimer laser therapy after CEA.

## Conflicts of interest

Drs Kato, Toriyama, Sato, and Morita were involved in a clinical trial of cultured epidermal autograft containing melanocytes for vitiligo patients by Japan Tissue Engineering Co, Ltd. Drs Tanaka and Inoie are employees of the company.
